# The search for pain biomarkers in the human brain

**DOI:** 10.1093/brain/awy281

**Published:** 2018-11-20

**Authors:** André Mouraux, Gian Domenico Iannetti

**Affiliations:** 1 Institute of Neuroscience, UCLouvain, Brussels, Belgium; 2 Department of Neuroscience, Physiology and Pharmacology, University College London, London, UK; 3 Neuroscience and Behaviour Laboratory, Istituto Italiano di Tecnologia, Rome, Italy

**Keywords:** pain, neuroimaging, EEG, fMRI, biomarkers

## Abstract

Non-invasive functional brain imaging is used more than ever to investigate pain in health and disease, with the prospect of finding new means to alleviate pain and improve patient wellbeing. The observation that several brain areas are activated by transient painful stimuli, and that the magnitude of this activity is often graded with pain intensity, has prompted researchers to extract features of brain activity that could serve as biomarkers to measure pain objectively. However, most of the brain responses observed when pain is present can also be observed when pain is absent. For example, similar brain responses can be elicited by salient but non-painful auditory, tactile and visual stimuli, and such responses can even be recorded in patients with congenital analgesia. Thus, as argued in this review, there is still disagreement on the degree to which current measures of brain activity exactly relate to pain. Furthermore, whether more recent analysis techniques can be used to identify distributed patterns of brain activity specific for pain can be only warranted using carefully designed control conditions. On a more general level, the clinical utility of current pain biomarkers derived from human functional neuroimaging appears to be overstated, and evidence for their efficacy in real-life clinical conditions is scarce. Rather than searching for biomarkers of pain perception, several researchers are developing biomarkers to achieve mechanism-based stratification of pain conditions, predict response to medication and offer personalized treatments. Initial results with promising clinical perspectives need to be further tested for replicability and generalizability.

## Teaser

Do current neuroimaging-based biomarkers developed to ‘objectively’ assess pain perception truly relate to pain? Mouraux and Iannetti critically review the evidence, and examine the utility of brain biomarkers for achieving mechanism-based patient stratification, predicting treatment responses and offering personalized treatments.

## Introduction

Physical pain is intrinsically unpleasant and aversive. This is the very reason why it is advantageous for survival: it drives behaviours that avoid bodily injury when interacting with the environment. Yet, especially in modern societies, acute pain is often devoid of behavioural advantage. Think, for example, of the pain experienced during medical interventions. Furthermore, an increasing number of individuals suffer from pain that lasts for months or years (Breivik *et al.*, 2006). This chronic pain is not only lacking any obvious behavioural benefit (Hodges and Tucker, 2011), but also heavily impairs quality of life. The fact that pain has a major negative impact on human wellbeing is often used as a persuasive argument to justify the funding of pain research. The prospect, which has shaped the way many pain neuroscientists conceive, design and interpret their work, is that beyond improving basic knowledge of the neural mechanisms of sensory perception, research in the field of pain will also lead to the development of more effective means to treat pain and reduce suffering.

Three arguments are usually set forth to uphold this prospect in the field of pain neuroimaging (Kupers and Kehlet, 2006; Borsook *et al.*, 2007, 2010; de Vries *et al.*, 2013; Lee and Tracey, 2013; Morton *et al.*, 2016; Grosen *et al.*, 2017; Tracey, 2017). First, it is often claimed that functional neuroimaging could be used to derive brain biomarkers that measure pain ‘objectively’. This would provide a solution to the hurdle of assessing pain using verbal reports, which are considered to be inherently prone to response biases (Wager *et al.*, 2013; Kumbhare *et al.*, 2017). Such biomarkers for pain would make it possible to quantify pain severity and the effects of treatments in an objective and undisputable ‘evidence-based’ fashion. Second, it is postulated that a mechanism-based diagnosis of clinical pain conditions is essential for adequate pain management (Woolf and Max, 2001; Woolf, 2008; Borsook *et al.*, 2010, 2011; Lee and Tracey, 2013). By disclosing the neural mechanisms underlying pain in individual patients, neuroimaging could thus improve clinical diagnosis and care, for example, by predicting individual response to treatment (Wartolowska and Tracey, 2009; Denk *et al.*, 2014; Tetreault *et al.*, 2016; Davis and Seminowicz, 2017; Kumbhare *et al.*, 2017). Third, it has been proposed that functional neuroimaging and electrophysiology could be used to quickly identify new pain-relieving drugs by characterizing their effects on CNS pain ‘circuits’ (Woolf and Max, 2001; Martucci *et al.*, 2014), an approach sometimes referred to as ‘pharmaco-fMRI’ or ‘pharmaco-EEG’ (Schweinhardt *et al.*, 2006; Wise and Tracey, 2006; Woolf, 2008; Gram *et al.*, 2013).

**Figure 1:**
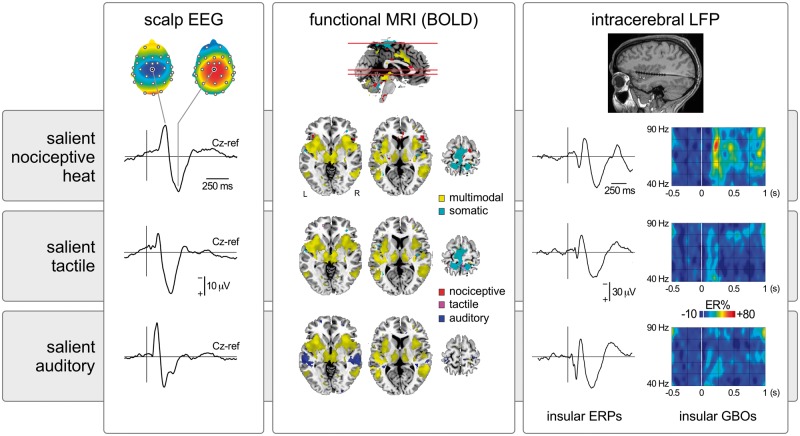
**Transient nociceptive stimuli causing pain.** In this example heat laser pulses delivered to the right hand (scalp EEG and intracerebral LFP) or foot (functional MRI) elicit large-scale brain responses. In scalp EEG, the response is dominated by a large negative-positive wave maximal at the scalp vertex (electrode Cz), probably originating from bilateral operculo-insular regions, the cingulate cortex and, possibly, the contralateral primary somatosensory cortex. Responses in similar regions are also detected using functional MRI. Importantly, equally salient but non-painful and non-nociceptive tactile or auditory stimuli elicit very similar EEG and functional MRI responses, indicating that most of this activity is unspecific for pain or nociception and, instead, multimodal (Mouraux and Iannetti, 2009; Mouraux *et al.*, 2011). Similarly, although the insula has been proposed to be strongly involved in pain perception, equally salient nociceptive and non-nociceptive stimuli trigger similar local field potentials (LFPs) recorded directly within the insula (Liberati *et al.*, 2016). Nevertheless, other less prominent features of the sampled activity might be more selective for pain or nociception, as reflected by the selective increase of gamma-band oscillations (GBOs) when painful heat stimuli are presented (Liberati *et al.*, 2018). BOLD = blood-oxygen level-dependent; ER% = event-related change in oscillation amplitude; ERP = event-related potential.

One important question challenges the use of functional neuroimaging to derive ‘biomarkers’ of pain perception: does the brain activity sampled by these techniques when an individual experiences pain correspond to the neuronal activity causing the emergence of the painful percept? As summarized in a review paper that we published a few years ago (Iannetti and Mouraux, 2010), we and others (Carmon *et al.*, 1976; Chapman *et al.*, 1981; Melzack, 1999; Downar *et al.*, 2003) have expressed concern regarding the specificity for pain of the brain responses classically observed when experiencing transient pain, i.e. the so-called ‘pain matrix’, a label covertly implying some specificity for pain. The concern is based on the observation that largely the same functional neuroimaging responses can be elicited by non-painful stimuli, provided that they are salient enough (Chapman *et al.*, 1981; Downar *et al.*, 2003; Mouraux and Iannetti, 2009; Mouraux *et al.*, 2011) (Fig. 1). More recently it was also shown that a virtually identical ‘pain matrix’ response can be observed in patients with congenital insensitivity to pain (Salomons *et al.*, 2016), thus providing further evidence that these brain responses are largely non-specific for pain. (This statement does not imply that neural activities specific for pain do not exist. Instead, it implies that the neural activities captured by current EEG or functional MRI techniques, which reflect synchronous activity within large populations of neurons, are—at the very least—largely unspecific for pain.) To escape from these controversies, many researchers now refrain from using the term ‘pain matrix’, and opt instead for terms like ‘pain network’, ‘pain signature’ or ‘neural circuits’ (Tracey and Mantyh, 2007; Seifert and Maihofner, 2011; Lelic *et al.*, 2012; Longo *et al.*, 2012; De Simone *et al.*, 2013; Wager *et al.*, 2013). Such labels are equally suggestive of the idea that the brain responses that are being measured reflect neural activity somehow unique for pain. To elaborate on only one of these examples, the term ‘signature’ denotes a distinctive pattern, product or characteristic by which something can be unequivocally identified. As detailed below, we argue that the attempts to falsify the hypothesis that the brain responses being measured are specific for pain using appropriate control stimuli have been insufficient, and the liberal use of terms implying specificity has biased the interpretation of several pain neuroimaging results.

The danger of assuming that brain responses sampled when experiencing pain are specific for pain is well illustrated by the way several pain neuroimaging results have been communicated by the general media. For example, a press release reporting a neuroimaging study on pain in infants conducted by Goksan *et al.* (2015) stated that because the ‘brains of babies light up in a very similar way to adults when exposed to a painful stimulus, new-borns experience pain in the same way as adults’ (http://www.ox.ac.uk/news/2015-04-21-babies-feel-pain-‘-adults’). Evidently, this conclusion, based on reverse inference, is valid if and only if the observed brain activity is specific for pain, as detailed in the ‘Pain-specific and pain-selective brain activity’ section below.

In the following sections, we first examine whether an established assumption—that there is a real clinical need for an ‘objective’ laboratory measure for the subjective perception of pain—is truly justified. Second, we examine the issue of pain specificity of the brain activity sampled using functional neuroimaging and electrophysiological techniques. This is necessary and timely, given the increasing use of new methods to analyse brain activity such as multivariate pattern analysis of functional MRI data to reveal ‘pain signatures’ (Wager *et al.*, 2013), as well as the proposal of new theoretical concepts such as the ‘pain connectome’ (Kucyi and Davis, 2015, 2017), in which pain would emerge from widespread brain network activity. Third, we assess pragmatically whether current biomarkers derived from neuroimaging have the ability to measure pain ‘objectively’. Finally, we evaluate the strength of the evidence supporting the use of functional neuroimaging to perform mechanism-based stratification of patients with chronic pain, predict response to treatment, and assist the pharmacological development of novel treatments for pain.

## Are neuroimaging biomarkers for pain really useful?

One of the enticing prospects of functional neuroimaging is that the sampled brain activity can be related to certain perceptual states. A spectacular example of what advanced algorithms to analyse brain activity can achieve is the decoding of the content of visual perception in wakefulness and sleep using brain activity sampled in visual areas (Horikawa *et al.*, 2013). This gives strong theoretical and technological hope for the ability to use functional neuroimaging to decode the occurrence of a wide range of perceptions, including pain.

A crucial point to consider when one aims to use neuroimaging to ‘measure’ pain is the fact that, such as any other percept, pain is an intrinsically subjective experience. Therefore, even though measures of brain activity can be objective from a physiological standpoint (e.g. an objective measure of cerebral blood flow or scalp potential), biomarkers derived from these measures will be truthful correlates of perceived pain if and only if they account for the subjectivity of the pain experience (Robinson *et al.*, 2013; Sullivan *et al.*, 2013).

Healthcare providers often question the clinical utility of using complex and expensive neuroimaging techniques as means to measure pain because just interrogating patients about their pain is much simpler and more straightforward (Robinson *et al.*, 2013). In the vast majority of circumstances, it is hard to argue that they are not right. One advantage of using a measure of brain activity to assess pain could be to bypass verbal reports, as these could be influenced by factors other than the experienced pain, and not only in the context of malingering. For example, patients may consciously or unconsciously exaggerate their report of pain to attract the attention of the caregiver or make sure that their complaint is taken into consideration. Other reasons may lead patients to understate their pain experience, such as not wanting to seem weak or be a nuisance, or satisfy the caregiver following a treatment. Finally, physicians can also be subject to biases, for example in relation to culture and ethnicity (Hoffman *et al.*, 2016). Therefore, contextual factors are not only important determinants of the subjective pain experience, but might also modulate how the pain experience is reported and evaluated, and this is probably why some scientists and physicians have been seeking more ‘objective’ ways to measure pain. However, although verbal reports collected in large samples and controlled experimental settings may be more reliable than individual reports in clinical settings, the only way to test the sensitivity and specificity of alternative measures of pain perception is to compare them to verbal reports.

**Figure 2:**
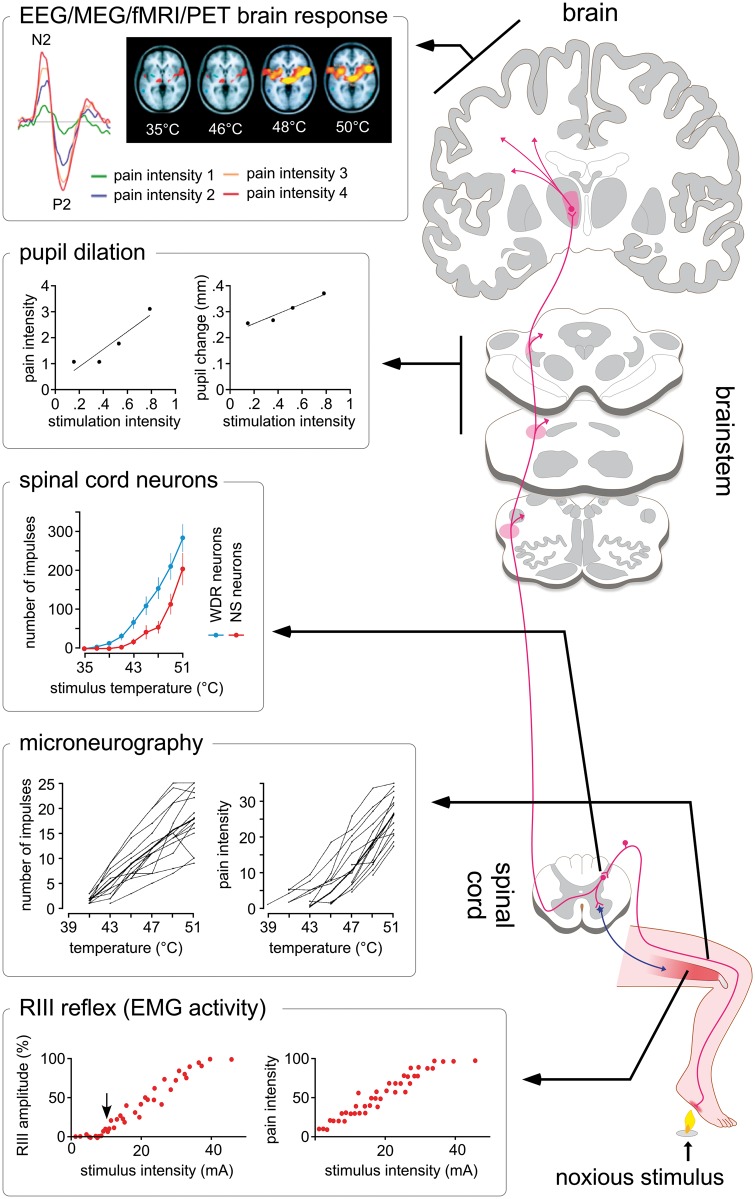
**Several physiological measures can, in some circumstances, correlate reliably with the reported intensity of perceived pain.** As shown in the *left* plots, these measures can be obtained at different levels of the neuraxis: peripheral nociceptor activity recorded using microneurography [firing rate of a peripheral C-fibre nociceptor and intensity of perception as a function of stimulation temperature; adapted from Torebjork *et al.* (1984)], spinal cord activity assessed using dorsal horn electrophysiology in animals [firing rate of WDR and nociceptive specific dorsal horn neuron as a function of stimulation temperature; adapted from Khasabov *et al.* (2001)] or the recording of nociceptive RIII reflex activity using EMG in humans [amplitude of the RIII nociceptive withdrawal reflex in the tibialis anterior and intensity of perception as a function of the intensity of electric stimulation of the sural nerve; adapted from Willer *et al.* (1984)], cortical activity sampled using non-invasive functional neuroimaging techniques such as EEG [event-related brain potentials elicited by laser heat stimulation of the hand dorsum as a function of intensity of perception; adapted from Iannetti *et al.* (2008)], magnetoencephalography (MEG), functional MRI [fMRI-BOLD response elicited by thermal stimulation at different target temperatures; adapted from Bornhovd *et al.* (2002)] or PET, but also autonomic responses such as pupil dilation [magnitude of pupil dilation and intensity of perception as a function of stimulation intensity; adapted from Chapman *et al.* (1999)].

In few specific instances, means to assess the presence or intensity of pain that do not rely on verbal reports would be undeniably useful. First, to demonstrate pain in patients seeking compensation in a medico-legal context (Reardon, 2015; Salmanowitz, 2015; Davis *et al.*, 2017), because the financial incentive for appearing disabled by pain leads indemnitors to question the sincerity of verbal reports. Second, to assess pain or nociception in individuals that are unable to communicate, such as infants (Goksan *et al.*, 2015; Hartley *et al.*, 2017), adults with consciousness disorders (Boly *et al.*, 2008), cognitive impairment (Defrin *et al.*, 2015), and patients under general anaesthesia (Cowen *et al.*, 2015). However, it should be stressed that the physiological properties of the brain of such patients (properties that determine the signal measured using functional neuroimaging and electrophysiology) can be different from those of the normal adult brain (Iannetti and Wise, 2007; Marshall *et al.*, 2014). Hence, pain biomarkers derived from healthy adult volunteers are not necessarily valid to assess pain in these clinical conditions. Furthermore, the utility of neuroimaging-based pain biomarkers should be compared with that of biomarkers using pain-related behaviours or physiological responses that may be easier to measure and implement in a clinical setting, such as changes in facial expression, heart rate, pupil diameter or skin conductance (Cowen *et al.*, 2015) (Fig. 2).

## Pain-specific and pain-selective brain activity

A great number of studies have shown that noxious experimental stimuli perceived as painful elicit activity within a wide array of brain regions including the primary (S1) and secondary (S2) somatosensory cortices, the insula and the anterior cingulate cortex (ACC) (Peyron *et al.*, 2000; Garcia-Larrea *et al.*, 2003; Apkarian *et al.*, 2005; Bushnell and Apkarian, 2005; Tracey and Mantyh, 2007). Because these responses (i) are consistently observed when subjects are experiencing acute pain; and (ii) often correlate with the amount of pain experienced by the subject, several studies have claimed that they reflect, just to cite a few examples, ‘the neural substrates of pain’ (Ploghaus *et al.*, 1999), the functioning of ‘cortical areas devoted to pain elaboration’ (de Tommaso *et al.*, 2005), or even ‘different aspects of pain elaboration’ (Di Clemente *et al.*, 2013). As discussed below, these conclusions do not consider the exclusivity of the relationship between these responses and the state of experiencing pain (Poldrack, 2006; Iannetti and Mouraux, 2010).

A first question that arises is what is meant by ‘pain-selective’ and ‘pain-specific’. When these terms are used to qualify the functional significance of neural activity, they are often used interchangeably. Looking at how they are used in other domains is informative, because it indicates that the two terms in fact convey different meanings. In pharmacology, compound A is qualified as more selective than compound B, if A has a greater effect than B on the target population of cells or receptors, and a smaller effect than B on the non-target populations of cells or receptors. Similarly, in analytical chemistry, a method is qualified as more selective if it can quantify an analyte with less interference from other components (Vessman, 1996). A parallel can also be made between the concept of selectivity in pharmacology and the concept of response preference in single unit electrophysiology, which indicates neurons that respond preferentially to a given stimulus, although they also respond to other types of stimuli. Therefore, a neuron selective for pain would exhibit a response preference for pain, i.e. it would fire more strongly when pain is present as compared to when pain is absent. It follows that selectivity is not an all-or-nothing property and, instead, can be graded or quantified.

In contrast, the term specificity implies an all-or-nothing characteristic. In pharmacology, a molecule specific for a given target would have an exclusive effect on that target and no effect at all on any other target. In analytical chemistry, a specific assay would be one that quantifies an analyte without any interference from other components (Vessman, 1996). It follows that a ‘pain specific’ neuron would be a neuron that increases its firing rate when pain is present, and never does when pain is absent, i.e. it would exhibit the highest degree of selectivity. Strictly speaking, demonstrating that a neural response is specific for pain is practically impossible: it would require testing the entire spectrum of stimuli and conditions that could produce a response, to show that the response is observed only in the particular cases where pain is experienced. For this reason, we argue that the terms pain-specific or nociceptive-specific should be avoided not only because it is practically impossible to demonstrate specificity, but also because it is more informative to approach the problem in probabilistic terms and try to assess the likelihood that a given response is preferential for pain, i.e. its selectivity. This requires determining the probability of pain being present when the neural response is observed. Crucially, this is not the same as determining the probability of observing the neural response when pain is present, as it will also depend on the probability of the neural response being observed when there is no pain (Senn, 2013).

A parallel can be made between the definition of specificity and the validity of conclusions based on reverse inference of functional neuroimaging data (Poldrack, 2006). Stating that a given brain response is a specific ‘signature’ for a given mental state or sensation requires demonstrating that this brain response is not only always observed when that mental state is present, but also that it is never observed in any conditions where that mental state is absent. Because it is not possible to test the infinite number of conditions where a mental state is absent, one can judge the likelihood of the statement being true to be high if the exclusivity of the relationship is demonstrated in many conditions (Iannetti *et al.*, 2013).

When reflecting on the criteria necessary to consider a neural response as selective or specific for pain, it is also important to consider the perceptual qualities that are coupled with pain, as defined by the International Association for the Study of Pain (IASP): ‘an unpleasant sensory and emotional experience associated with actual or potential tissue damage, or described in terms of such damage’ (Merskey and Bogduk, 1994). Thus, pain is not defined by a unique attribute, but by the conjunction of several attributes, such as the intrinsic unpleasantness of the experience and its threatening association with bodily damage. In addition, in a large number of circumstances, pain is also highly salient and, thus, attracts attention. Because pain signals a threat for the body, it also has strong behavioural relevance. However, sensations that are unpleasant, salient and behaviourally-relevant are not necessarily painful (e.g. the hideous sound produced when fingernails scrape a blackboard). Therefore, when assessing the selectivity or specificity for pain of a given brain response, it is crucial to use non-painful control stimuli that elicit sensations matched with respect to unpleasantness, salience and relevance. These different aspects are strongly associated with pain but they do not capture what truly distinguishes physical pain from other sensations, i.e. the experience of actual or potential tissue damage (Merskey and Bogduk, 1994). If the responses to painful stimuli are not compared to non-painful control stimuli that elicit sensations matched with respect to unpleasantness, salience and relevance, it follows that it is impossible to determine whether the differences observed between the brain responses elicited by painful and non-painful stimuli reflect neural activities that are selective for pain, or neural activities that are selective for these other features.

**Figure 3:**
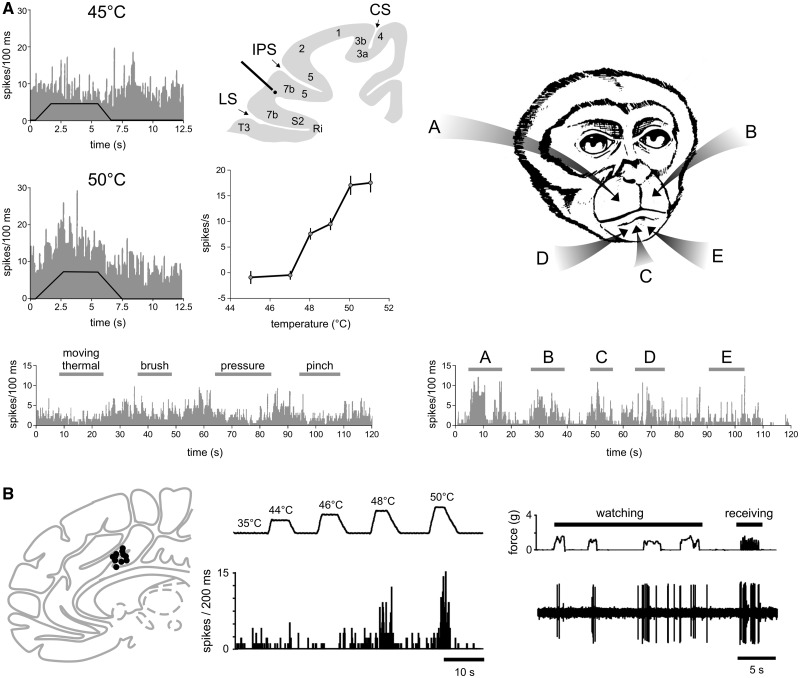
**Assessing the selectivity for pain of a given brain response requires not only to demonstrate that the response is present when pain is experienced, but also to demonstrate that it is not present when pain is not experienced.** (**A**) Dong *et al.* (1994) recorded the activity of a single neuron located in area 7b, close to the secondary somatosensory cortex. When tested with a variety of thermal and mechanical somatic stimuli, the neuron responds to noxious heat stimuli applied to the face in a graded fashion, whereas it does not respond to a variety of mechanical stimuli. Based on these observations one might be tempted to conclude that the neuron is specific for burning pain. However, the same neuron also responds vigorously to visual stimuli approaching its receptive field (A–E), and the response was most prominent when the approaching object was novel or threatening. Therefore, in classical pain studies testing the response properties with noxious and innocuous somatosensory stimuli, this neuron would be labelled as nociceptive-specific (NS). However, this labelling would be incorrect, at least until the lack of responses to a wide range of equally salient, unpleasant and behaviourally-relevant stimuli has been comprehensively demonstrated. (**B**) Similarly, Hutchison *et al.* (1999) performed single unit recordings in the human anterior cingulate cortex, and found many neurons responding to noxious heat stimuli, and not to slow-rising innocuous mechanical stimuli. However, some of these neurons also responded to watching noxious stimuli being delivered to the experimenter, suggesting that they may serve a supramodal function related or consequent to stimulus saliency or threat detection. CS = central sulcus; IPS = intraparietal sulcus; LS = lateral sulcus. Adapted with permission from Fig. 7 in Dong *et al.* (1994) and Fig. 1 in Hutchison *et al.* (1999).

Does the brain contain neurons specific or selective for pain? At present, there is no clear answer to this question. Using anterograde trans-synaptic tracing from the spinal cord via the thalamus to the cerebral cortex in monkeys, Dum *et al.* (2009) confirmed the existence of direct di-synaptic spino-thalamo-cortical projections to S1, S2, the insula and the ACC. However, the projections to S1 were very sparse (<5%) as compared to projections to the posterior insula (40%), S2 (30%) and the mid-region of the cingulate cortex (24%), which thus appear to constitute the three main cortical targets of nociceptive input. Single-unit recordings performed in anaesthetized and/or awake animals have identified neurons responding to nociceptive stimuli in all these areas (Robinson and Burton, 1980; Kenshalo and Isensee, 1983; Dong *et al.*, 1989; Sikes and Vogt, 1992; Yamamura *et al.*, 1996; Treede *et al.*, 1999; Dum *et al.*, 2009). However, a large proportion of these neurons show only a moderate selectivity for nociceptive stimuli, as they respond more strongly to nociceptive stimuli as compared to non-nociceptive somatic stimuli, but nevertheless respond clearly to both types of stimuli. These neurons are often referred to as ‘wide dynamic range neurons’ (WDR). Other neurons appear to have higher mechanical or thermal activation thresholds and, therefore, appear to exhibit a stronger selectivity for nociception. These are often labelled as ‘high threshold’ or ‘nociceptive specific’ neurons. Supporting the view that S1 is not a major target of spinothalamic input, neurons responding to nociceptive input in S1 are strikingly sparse (Kenshalo and Isensee, 1983; Kenshalo *et al.*, 2000). However, researchers have argued that this could be dependent on (i) whether the recordings are performed in anaesthetized versus awake animals; (ii) which subregions of S1 were sampled; and (iii) the type of nociceptive stimuli that are used to elicit responses (Mancini *et al.*, 2012; Vierck *et al.*, 2013; Jin *et al.*, 2018). Considering that tracing studies indicate S2 and the insula as main targets of spinothalamic input (Dum *et al.*, 2009), it is surprising that electrophysiological recordings identified only a very small number of neurons responding to nociceptive input in these regions. For example, Robinson and Burton (1980) reported, in awake monkeys, that most neurons in S2 and the insula respond to a variety of somatic stimuli, but almost never respond to noxious stimuli. An explanation for this discrepancy could be a searching bias: noci-responsive neurons might be confined to specific subregions of S2 and the insula, which were not explored by electrophysiological recordings. Supporting this interpretation, Dong *et al.* (1989) identified a cluster or noci-responsive neurons in the lateral sulcus on the upper bank of the parietal operculum, at the border between S2 and area 7b. Of 123 neurons responding to somatic stimuli, 118 responded to innocuous mechanical stimuli and only five responded exclusively to noxious mechanical stimulation. The authors were careful in their interpretation, stating that ‘additional testing is needed to support the tentative conclusion that an exclusive nociceptive-specific population exists’ in this region. In a subsequent study, Dong *et al.* (1994) attempted to further characterize the function of these noci-responsive neurons. In that study, 21 of 244 neurons responding to somatosensory stimulation responded to noxious heat stimuli, but only one neuron responded exclusively to noxious heat and mechanical stimuli. Importantly, approximately one-third of the neurons responding to noxious heat were at least bimodal: they also responded to spatially-aligned threatening or novel visual stimuli moving towards their cutaneous receptive fields. This finding indicates that the selectivity of these neurons does not relate to nociception but, instead, to threat, saliency, and/or behavioural relevance (Fig. 3).

As compared to S1 and the operculo-insular cortex, a greater number of neurons responding to nociceptive stimuli have been identified in the cingulate cortex (Sikes and Vogt, 1992; Yamamura *et al.*, 1996; Hutchison *et al.*, 1999), including a study conducted in humans (Hutchison *et al.*, 1999). However, a good amount of these noci-responsive neurons in the cingulate may, in fact, be supramodal. For example, Sikes and Vogt (1992) found that out of 542 units recorded in area 24 of the cingulate cortex of rabbits, 150 units responded to noxious electrical stimulation of the skin. Of 221 units tested with noxious mechanical stimuli, only 91 responded, and of 47 units tested for noxious heat stimulation only nine responded. Approximately 25% of the units that responded to noxious mechanical stimulation also responded to noxious heat stimulation. These noci-responsive neurons did not respond to slow-rising innocuous mechanical stimuli, such as light stroking, brushing or pressure, an observation suggesting selectivity or specificity for nociception. However, more than half of these units did respond to fast-rising innocuous mechanical stimuli, such as tapping of the skin (11/19 tested units), suggesting that these ‘noci-responsive’ neurons in the ACC might in fact be more sensitive to the suddenness or salience of the stimulus. Similarly, Hutchison *et al.* (1999) performed single-unit recordings of the ACC in 11 human patients. In four patients, 11 ACC neurons responded to contralateral noxious thermal and/or noxious mechanical stimuli. None of these neurons responded to innocuous tactile stimuli. However, three of these 11 neurons also responded to anticipation or observation of potentially painful stimuli. Furthermore, the electrical stimulation of the ACC failed to elicit painful sensations, even at sites where noci-responsive neurons had been identified. Again, this suggests that at least a fraction of neurons responding to nociceptive stimuli may serve supramodal functions related or consequent to the detection of stimulus saliency, threat detection and/or behavioural relevance (Hutchison *et al.*, 1999).

Taken together, studies conducted so far suggest that if truly nociceptive-specific neurons or even neurons highly selective for nociception exist in the brain, they are very scarce (Wall, 1995). Furthermore, because these studies were almost exclusively conducted in animals that cannot report whether they are experiencing pain, how the activity of these neurons relates to the perception of pain is largely unknown. Finally, as we will argue below, the ability to perceive and discriminate pain from other somatic sensations does not necessarily require the activity of individual neurons with high selectivity for pain, because distinct percepts could emerge from distinct patterns of activity in neuronal populations having, individually, a low response selectivity.

Does the brain contain areas that are specific or selective for pain? This question asks whether the brain contains areas in which pain-selective or pain-specific neurons may be spatially segregated, in the same way as, for example, neurons whose primary function is to process visual input are clustered in the primary visual cortex, or neurons whose primary function is to generate somatic motor output are clustered in the primary motor cortex. The main distinction between this question and the previous question is that it addresses cortical specialization at population level, using techniques sampling the summated activity of large populations of neurons, such as functional MRI or EEG. As mentioned above and shown in Fig. 1, using such brain imaging techniques, there is an important overlap between the brain responses to acute pain and the brain responses that can be elicited by non-painful salient tactile, auditory or visual stimuli. There are at least two possible explanations for this overlap. First, it could be consequent to the difficulty in detecting fine-grained spatial differences in cortical activity using univariate group-level analyses of spatially-smoothed functional MRI data, or using low spatial resolution scalp EEG and MEG data. Second, it could be due to the fact that transient stimuli elicit widespread large amplitude saliency-related responses, which could conceal smaller amplitude activity specific for pain.

As detailed above, tracing studies performed in monkeys have shown that the main projection sites of spinothalamic input are the insula, S2 and the cingulate cortex (Dum *et al.*, 2009), suggesting that these areas play a primary role in the cortical processing of ascending nociceptive input. Whether these areas are selective for pain is a timely question, given recent claims that ‘the dorsal posterior insula subserves a fundamental role in human pain’ (Segerdahl *et al.*, 2015), or that the dorsal anterior cingulate cortex is ‘selectively involved in pain-related processes’ (Lieberman and Eisenberger, 2015).

Segerdahl *et al.* (2015) used functional MRI based on arterial spin labelling to identify sustained variations in brain activity whose time courses follow equally sustained changes in pain perception generated by the combination of topical capsaicin and heat. The originality of this tonic stimulation approach, which has been also used in a small number of other studies (Schulz *et al.*, 2015), is the attempt to move away from sampling responses triggered by the onset of transient noxious stimuli. The authors postulated that slow variations in brain activity that correlate with slow variations of pain intensity could isolate brain activity more directly related to the perception of pain. The strongest correlation was observed in the dorsal posterior part of the insula. This led the authors to conclude ‘a specific role for the dorsal posterior insula in pain’. The propensity for sensationalism of the general media led to a press report stating that the ‘ouch zone of the human brain had been identified’ (http://www.ox.ac.uk/news/2015-03-09-‘ouch-zone’-brain-identified-0). However, we and others argued that the experiment did not include appropriate control stimuli to substantiate the claim for ‘specificity’, or even ‘selectivity’. Indeed, presence versus absence of pain was by no means the only difference between their sustained pain condition and their control sustained touch condition (Davis *et al.*, 2015) (see http://f1000research.com/articles/4-362 for reviewer and reader comments). Another crucial difference was that the nociceptive stimulation was much more salient and unpleasant than the tactile stimulation, which in fact failed to elicit activity in S1. There is now strong experimental evidence indicating that the differences between the insular responses elicited by nociceptive and tactile stimulation observed by Segerdahl *et al.* (2015) could have been driven entirely by factors other than pain. For example, invasive intracerebral EEG recordings from the human insula showed that transient painful stimuli and non-painful tactile, auditory and visual stimuli elicit largely similar responses in all subregions of the human insula including the dorsal posterior insula, provided that they are similarly salient (Liberati *et al.*, 2016). Furthermore, there is a case report of a patient with bilateral extensive damage to the insula with intact abilities to experience and express pain (Salomons *et al.*, 2016). Similarly, although some studies suggested that lesions of the insula may impair the ability to perceive pain (Garcia-Larrea *et al.*, 2010), a later review of 24 patients with acute unilateral stroke lesions primarily affecting the insular cortex reported no changes in cold pain, heat pain or mechanical pain thresholds (Baier *et al.*, 2014). Nevertheless, some studies have suggested that lesions of the insula impair the ability to perceive pain and that direct cortical stimulation of the insula or epileptic activity in the insula can generate pain, although only in rare cases (Isnard *et al.*, 2011; Mazzola *et al.*, 2012). Furthermore, intracerebral recordings of local field potentials in the human insula have shown that painful heat stimuli elicit gamma-band oscillations that are not observed in response to similarly salient tactile, auditory or visual stimuli (Liberati *et al.*, 2018) (Fig. 1). Clearly, in the current state of affairs, whether or not the insula plays a causal role in the generation of pain remains a largely open question.

Another study recently claimed that the dorsal anterior cingulate is ‘selective for pain’ (Lieberman and Eisenberger, 2015). The claim stems from a meta-analysis conducted using Neurosynth, a tool to analyse a database of published functional neuroimaging data based on the frequency of terms used in the manuscripts reporting that data. The authors found (i) that activation of the dorsal anterior cingulate is more consistently reported in publications using pain-related terms (‘pain’, ‘painful’, ‘noxious’) as compared to publications not using these terms; and (ii) that this is not the case for studies that frequently used terms related to executive control (‘executive’, ‘working memory’, ‘effort’, ‘cognitive control’, ‘cognitive’, ‘control’), conflict processing (‘conflict’, ‘error’, ‘inhibition’, ‘stop signal’, ‘Stroop’, ‘motor’), or salience (‘salience’, ‘detection’, ‘task relevant’, ‘auditory’, ‘tactile’, ‘visual’). The validity of this comparison has been extensively critiqued elsewhere (Wager *et al.*, 2016; Yarkoni, 2018). Most importantly, using the same database but manually identifying the topics of the studies based on the title and abstract, Wager *et al.* (2016) showed that activation of the dorsal anterior cingulate was associated with a 12% probability of that study involving pain, on par with language (8%), emotion (12%), attention (19%) and memory (12%); indicating that the functional MRI response in the dorsal anterior cingulate is largely unselective for pain (see also Shackman *et al.*, 2011).

In summary, even though the operculo-insular and cingulate cortex appear to be the main cortical targets of inputs ascending the spinothalamic tracts (Dum *et al.*, 2009), the actual involvement of these two brain structures in generating painful percepts remains to be warranted and clarified.

Does the brain generate patterns of activity that are specific or selective for pain? Faced with the increasing evidence that pain does not appear to emerge from the activation of ‘pain-specific’ neurons or brain areas, several researchers have proposed that the experience of pain could emerge from the interactions between a population of interconnected neurons (Norrsell *et al.*, 1999; Melzack, 2005; Kucyi and Davis, 2015). In this view, which is also increasingly considered in other fields of neuroscience (Sporns, 2013), specificity or selectivity of single neurons or of single brain areas would not be required to generate qualitatively unique experiences, like pain. The opposing ‘labelled lines’ and ‘pattern’ theories of neural coding have nourished scientific debate for decades (Doetsch, 2000). The pattern coding theory was first proposed in the beginning of the 19th century as a solution to the problem that photoreceptors only sensitive to three colours of light can convey information about the entire spectrum of light colours. This idea was later extended to all sensory, motor and cognitive brain functions (Erickson, 1963). In pain research, supporters of the ‘specificity theory of pain’ have advocated that pain is a specific modality with its own receptors and pathways, i.e. pain-specific labelled lines; whereas defenders of the ‘pattern theory of pain’ proposed that pain results from the pattern of activation generated in receptors and pathways that can also generate non-painful percepts and, therefore, are unspecific for pain (Erickson, 1973; Norrsell *et al.*, 1999; Prescott *et al.*, 2014).

This view that pain could emerge from a specific distributed pattern of neural activity constitutes one of the rationales for using multivariate approaches to explore functional neuroimaging data obtained when subjects are experiencing pain. In contrast to univariate approaches, multivariate pattern analysis (MVPA) attempts to link a particular mental state, such as experiencing pain, with a specific spatial pattern of brain activity sampled with functional MRI or EEG (Schulz *et al.*, 2012; Wager *et al.*, 2013; Corradi-Dell‘Acqua *et al.*, 2016; Lindquist *et al.*, 2017; Woo *et al.*, 2017). However, it is crucial to emphasize that testing whether a given spatial pattern of brain activity constitutes a ‘pain signature’ requires exactly the same evidence that is needed to demonstrate the existence of ‘pain-specific’ neurons or brain areas. In addition to showing that the identified spatial pattern is always present when one experiences pain, one must also show that the spatial pattern of activity is never present in the absence of pain. Wager *et al.* (2013) found that the same spatial pattern of brain activity can be observed in a variety of conditions where subjects are experiencing physical pain, and labelled this pattern ‘neurological pain signature’ (Wager *et al.*, 2013). The authors also assessed the selectivity of this response by showing that it is not observed in a number of control conditions. However, they restricted their testing to control conditions that differed from the painful conditions in many ways other than the presence versus absence of pain. Specifically, their control conditions were either less salient, less behaviourally relevant, and/or not somatic; for example, a low-salience mild warm stimulus versus a high-salience burning heat stimulus, or a non-somatic ‘social pain’ stimulus versus a somatic painful stimulus. For this reason, it could well be that the spatial pattern of brain activity that they referred to as a ‘neurological pain signature’ was, in fact, a spatial pattern of brain activity that is selective for the occurrence of a salient somatic stimulus, regardless of whether it elicits pain. Significantly, in a later study, the same authors observed that the neurological pain signature fails to predict variations in pain induced by cognitive ‘self regulation’, i.e. by imagining that nociceptive stimuli are more painful or less painful than they are, thereby demonstrating that the neurological pain signature does not necessarily track the subjective pain experience (Woo *et al.*, 2015).

Within the framework that specific sensations could emerge from the interactions within a network of interconnected neurons, the interesting notion of a ‘dynamic pain connectome’ has been recently proposed (Kucyi and Davis, 2015, 2017). In this theoretical model, pain would be encoded in ‘the spatiotemporal signature of brain network communication that represents the integration of all cognitive, affective, and sensorimotor aspects of pain’ (Kucyi and Davis, 2015). At first glance, this view is highly similar to the one proposed by Melzack (1999, 2005) in the ‘neuromatrix theory of pain’, in which pain would be ‘a multidimensional experience produced by characteristic neurosignature patterns of nerve impulses generated by a widely distributed neural network in the brain’. The difference between the two theories is that the ‘connectome’ of Kucyi and Davis (2015) emphasizes that the specificity of the patterns is not only defined by which elements are part of the network, but also by the temporal characteristics of the activity generated within the different elements. The fact that there must be specific features of brain activity that underlie pain sensations is unquestionable, unless one takes a dualistic stance on the mind-body relationship. However, this is not sufficient to demonstrate that there is a ‘pain connectome’. Future work is needed to translate this general concept into a set of falsifiable hypotheses. Importantly, one must bear in mind that current functional neuroimaging techniques may not have the spatial and/or temporal resolution required to discriminate the potentially subtle spatio-temporal features of brain activity that underlie pain sensations.

## What can we conclude from the ability of neuroimaging biomarkers to measure pain?

When neuroimaging is used as a clinical tool to predict the intensity of pain perceived by a human subject, whether the brain activity used to ‘decode’ pain intensity reflects neural processes that are specific or selective for pain is not an issue (Hu and Iannetti, 2016). Indeed, to achieve this very pragmatic objective, the only requirement is that the index derived from the measured brain activity must vary systematically with pain intensity. Furthermore, the relationship between this index and pain intensity should not be influenced by factors not affecting pain perception, at least in the situations for which the ‘pain biomarker’ is intended to be used.

Reflecting on the use of functional neuroimaging to guess whether a subject is experiencing pain brings us to the meaning and definition of the term ‘specificity’ in the context of a clinical diagnostic test, which is radically different from when the term specificity is used to qualify the function of neuronal activity (see ‘Pain-specific and pain-selective brain activity’ section). When referring to a diagnostic test, specificity indicates the proportion of patients not affected by a given condition that are correctly identified as such (i.e. the rate of ‘true negatives’). Conversely, the term sensitivity refers to the proportion of patients affected by a given condition that are correctly identified as positive (i.e. the rate of ‘true positives’). Thus, when applied to a neuroimaging biomarker for pain, the terms specificity and sensitivity refer to the ability of that biomarker to correctly identify the absence of pain in patients without pain and to correctly identify the presence of pain in patients with pain, respectively. Demonstrating that a given pain biomarker has a high sensitivity and specificity does not necessarily imply that the neural activity from which that pain biomarker is derived corresponds to the neural activity specific for pain, i.e. neural activity causing pain. Thus, if a given neuroimaging index has a good specificity with respect to its ability to measure pain, it is incorrect to automatically assume that the neural activity from which the index is derived corresponds to the neural activity generating pain. In other words, a pain biomarker can have utility (if it has enough selectivity and sensitivity in a clinical setting, or it is useful as a dependent measure in an experimental setting), but this utility does not necessarily mean that the biomarker is of any theoretical interest or that we are any closer to understanding the biological conditions that are necessary and sufficient for the subjective experience of pain.

It is instructive to consider the large number of biological measures that can be used to measure pain, without having any causal role in generating pain (Fig. 2). For example, noxious stimuli cause phasic dilation of the pupil (Chapman *et al.*, 1999; Eisenach *et al.*, 2017). Even though the magnitude of pupil dilation often correlates with pain reports (and pupil dilation has indeed been proposed as an objective measure of pain; Cowen *et al.*, 2015), this would not lead anyone to conclude that pain is caused by the pupil dilation itself. Another example more directly related to the brain is the following. If one could properly sample the activity of all retinal ganglion cells or primary visual cortex neurons, it would be possible to reconstruct fairly accurately what an individual is seeing. However, this does not mean that the sampled activity is what creates the visual percept. It only means that the sampled activity contains information related to what is being seen. In other words, one could have an effective pain biomarker using neural activity that does not actually generate the painful percept, such as the activity of primary nociceptive afferents or dorsal horn neurons, or the activity of neurons related to processes that are consequential to the percept (Fig. 2).

As a matter of fact, this same issue has led to heated debates among researchers searching for the neural correlates of consciousness. Identifying these neural correlates often relies on comparing conditions in which consciousness is present to conditions in which consciousness is absent (e.g. the presentation of a supraliminal versus subliminal sensory stimulus). As emphasized by Aru *et al.* (2012), differences in brain activity observed across the two conditions do not necessarily highlight the neural activity giving rise to conscious percepts, because at least part of the observed differences could reflect a number of prerequisites and/or consequences of conscious processing (Aru *et al.*, 2012).

Another important concept relevant to the discussion about pain biomarkers is generalizability (Davis *et al.*, 2017). First, it is important to demonstrate that the biomarker generalizes beyond the dataset used to generate the biomarker. This requires that the dataset used to test the predictive power of the biomarker is not the same dataset used to generate the biomarker. Second, a given biomarker might have high sensitivity and specificity within the context in which it is developed, tested and used (e.g. presence versus absence of acute experimental pain) but a low sensitivity and specificity in other contexts (e.g. presence versus absence of sustained clinical pain), i.e. it might not generalize to all conditions where physical pain is experienced. For example, Huang *et al.* (2013) identified a biomarker derived from EEG activity that successfully allows predicting subjective pain reports to brief nociceptive heat stimuli, both within subjects and across subjects. This biomarker was based on the amplitude and latency of the negative and positive vertex waves typically evoked by fast-rising nociceptive thermal stimuli in the ongoing EEG. However, an important caveat—which Huang *et al.* (2013) clearly acknowledge—is that the relationship between the biomarker and subjective pain is not obligatory. We give three examples of a clear disruption of this relationship. First, simply repeating the fast-rising painful stimulus three times at a fixed 1-s interval has no effect on the intensity of the pain elicited by each of the three stimuli, but has a very strong effect on the magnitude of the stimulus-evoked brain potentials: compared to the EEG response to the first stimulus, the EEG responses to the second and third stimuli are markedly reduced (Iannetti *et al.*, 2008). In this situation, the biomarker described by Huang *et al.* would make the erroneous prediction that the second and third stimuli elicit less or no pain, thus producing a false negative. Second, compared to fast-rising nociceptive stimuli, slow-rising nociceptive stimuli can produce the same intensity of pain, but fail to produce a similarly large brain response, simply because the magnitude of these phase-locked EEG responses is strongly dependent on the phasic nature of the stimulus onset (Iannetti *et al.*, 2004; Baumgartner *et al.*, 2005). In this case, the biomarker proposed by Huang *et al.* (2013) would again underestimate the perceived pain and produce a false negative. Third, as we detailed above, several studies have shown that the brain responses elicited when experiencing acute pain are largely indistinguishable from the brain responses elicited by non-painful but saliency-matched tactile stimuli (Fig. 1) (Mouraux and Iannetti, 2009; Liberati *et al.*, 2016). In this case, the abovementioned pain biomarker would predict that individuals are experiencing pain when they are exposed to salient stimuli that are not painful.

**Figure 4:**
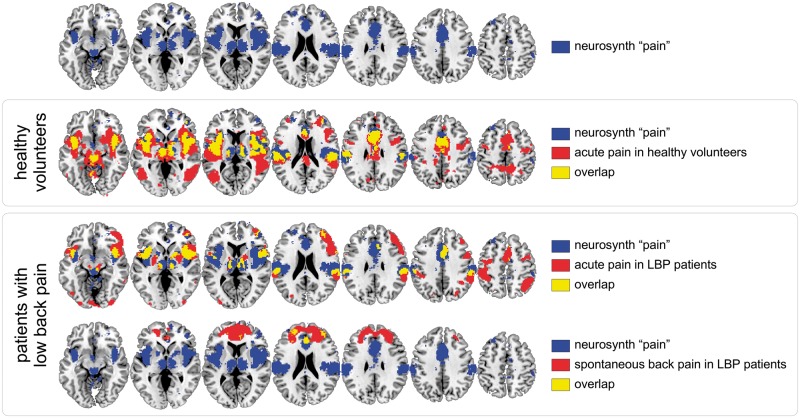
**Brain activity related to the perception of acute experimental pain and sustained clinical pain.** *Top* row shows the results of a meta-analysis of functional neuroimaging publications that frequently use the term ‘pain’ in the full text (Neurosynth-generated ‘reverse inference map’ using the term ‘pain’). The mask highlights the brain regions that are frequently often observed in ‘pain’ studies as compared to studies that do not frequently mention the term ‘pain’. The *second* row shows the overlap between this Neurosynth mask and the brain areas showing a significant BOLD response when transient painful stimuli are delivered to the right foot of healthy volunteers [data from Mouraux *et al.* (2011)]. Note the strong overlap, in yellow. The *third* row shows the BOLD response elicited by a transient painful stimulus in a group of patients with low back pain, generally similar to the BOLD response observed in healthy participants [data from Baliki *et al.* (2006)]. The *bottom* row shows, in the same patients, the regions where the BOLD signal correlates significantly with spontaneous fluctuations of low back pain. Note the lack of overlap between these areas and the Neurosynth-generated mask, indicating that a brain biomarker derived from brain activity triggered by acute painful stimuli is likely to be unable to assess pain in clinical conditions. LBP = low back pain.

Another issue is whether pain biomarkers derived from brain activity sampled in healthy participants experiencing acute pain can be used to assess pain in clinical conditions. Using functional MRI, Baliki *et al.* (2006) found that regions showing increased cerebral blood flow when human subjects experience acute pain are very different from the brain regions whose activity correlates with the spontaneous fluctuations of pain in patients with non-acute low back pain (Fig. 4). From this observation, it follows that a pain biomarker derived from the brain activity commonly observed when experiencing acute pain might have a good sensitivity and specificity to identify acute pain, but a very low sensitivity and specificity to identify the sustained pain frequently observed in clinical conditions. Obviously, this issue can be addressed by developing pain biomarkers derived from brain activity measured in patients experiencing clinical pain. In addition to Baliki *et al.* (2006), several other promising attempts have already been made in this direction. For example, Howard *et al.* (2011) and Hodkinson *et al.* (2013) used MRI arterial spin labelling to compare sustained measures of cerebral blood flow before versus after surgical tooth extraction, and found increased activity in the posterior and anterior insula, S2 and the anterior cingulate, i.e. a pattern of brain activity similar to the pattern of brain activity observed during acute pain. Using the same imaging technique, Wasan *et al.* (2011) compared patients with chronic low back pain in three conditions: a rest condition, a condition during which clinical manoeuvres were used to increase the intensity of back pain, and a third condition during which noxious heat was applied to the affected dermatome. They found that increasing the pathological back pain was associated with increased blood flow in somatosensory, prefrontal and insular cortices, and the superior parietal lobule. This pattern of brain activity differed from that observed during acute noxious heat pain, which showed no increase of activity in the superior parietal lobule. However, it also differed from the pattern of brain activity reported by Baliki *et al.* (2006). The differences could be due to the methodologies used to measure brain activity (blood-oxygen level-dependent signals versus arterial spin labelling), to create contrasts in clinical pain intensity (spontaneous fluctuations of clinical pain versus exacerbation of clinical pain using clinical manoeuvres), or to differences between the studied populations.

## Neuroimaging for mechanism-based diagnosis and stratification of patients with chronic pain

**Figure 5:**
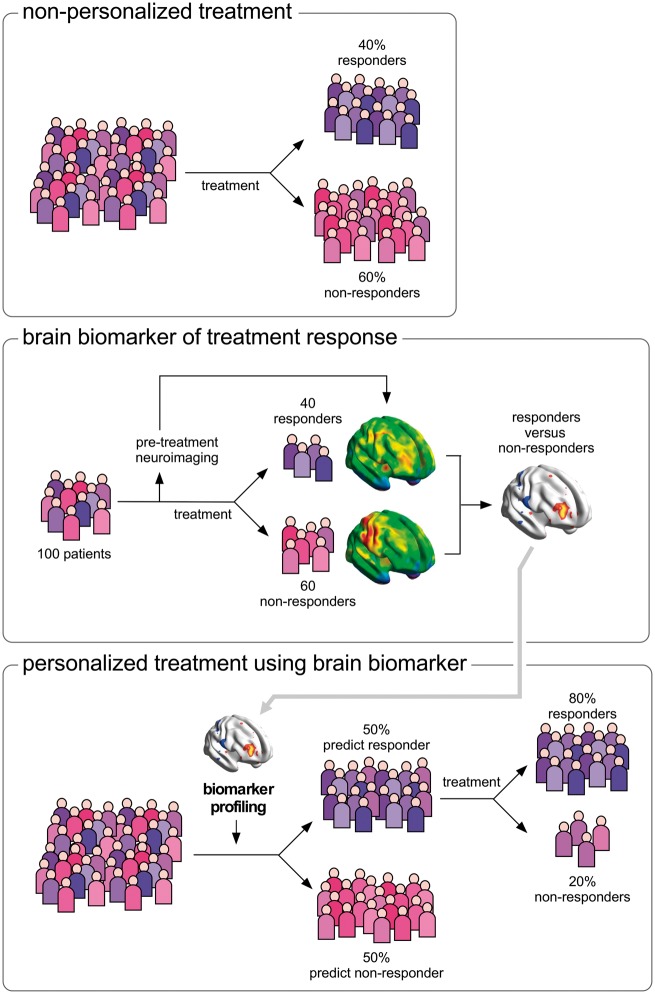
**Use of functional neuroimaging to predict treatment response and stratify patients.** Non-personalized treatment (*top*). When a population of patients is exposed to treatment X, some individuals respond to the treatment (e.g. 40%) and others do not (e.g. 60%). Brain biomarker of treatment response (*middle*). Functional brain imaging before any treatment is delivered to a group of patients to obtain predictive information about whether the patients are responders or non-responders. Personalized treatment using brain biomarker (*bottom*). The features of brain activity that distinguished responders from non-responders can be looked for in a new population of patients who have not yet been treated, to predict individual patient’s response, and thereby provide the first-line treatment that is most likely to be effective for that individual patient.

Researchers have suggested that it might be possible to use neuroimaging to identify different ‘constituent components’ or ‘networks’ underlying pain and its modulation (Di Clemente *et al.*, 2013; Denk *et al.*, 2014), such as the neural activities underlying central sensitization (Lee *et al.*, 2008), descending pain modulation (Bingel and Tracey, 2008), modulation of pain by emotions, attention or cognitive control (Peyron *et al.*, 2000; Kragel *et al.*, 2018), pain relief following a specific treatment (Iannetti *et al.*, 2005) or placebo analgesia (Bingel *et al.*, 2006; Schafer *et al.*, 2018). Thus, by identifying in individual patients suffering from chronic pain the engagement of different ‘constituent components’ contributing to the pain experience and its modulation, neuroimaging could make it possible to stratify patients in functionally distinct groups, with the exciting prospect of identifying which treatment is most likely to provide relief in individual patients and, hence, propose optimal first-line treatments (Fig. 5).

The idea that different measures of pain-related brain activity can be separated in functionally distinct components was probably first put forward by Albe-Fessard *et al.* (1985), when they proposed that pain processing can be separated into anatomically and functionally distinct ‘lateral’ and ‘medial’ pain systems, on the basis that ascending nociceptive inputs project onto lateral and medial thalamic nuclei, each having distinct cortical projections (Albe-Fessard *et al.*, 1985; Ingvar, 1999). In this model, the lateral pain system, comprising S1 and S2, would subserve the sensori-discriminative dimension of pain; whereas the medial pain system, comprising various brain structures including the cingulate cortex, would subserve the affective and cognitive dimensions of pain. However, although most researchers agree that this functional dichotomy between lateral and medial systems is an oversimplification (Brooks and Tracey, 2005; Iannetti and Mouraux, 2010), it is still used today as a framework to interpret experimental or clinical observations. For example, the finding that pain-evoked brain responses thought to originate from S2 are modulated by topiramate, a drug used as a pre-emptive treatment for migraine, led authors to conclude that the drug acts ‘specifically on the sensory-discriminative component of pain elaboration’ (Di Clemente *et al.*, 2013). Similarly, numerous studies on empathy and social exclusion have dichotomized pain-related brain activity as belonging either to ‘the affective part’ or the ‘sensori-discriminative part’, depending on whether they originate from brain structures belonging to the medial or lateral pain systems (Kross *et al.*, 2011, Novembre *et al.*, 2015). It should be noted that the empirical evidence supporting the functional distinction between ‘medial’ and ‘lateral’ pain systems is, to say the least, scarce. First, it relies on two experiments conducted by the same group, showing that the hypnotic modulation of pain unpleasantness is paralleled by a modulation of pain-evoked activity in the ACC, interpreted as a modulation of the ‘medial’ pain system (Rainville *et al.*, 1997), whereas hypnotic modulation of pain intensity is paralleled by a modulation of pain-evoked activity in S1, interpreted as a modulation of the ‘lateral’ pain system (Hofbauer *et al.*, 2001). Second, it relies on evidence from the single report of a patient with reduced ability to detect and discriminate pain, but preserved ability to experience its unpleasantness following a post-central lesion (Ploner *et al.*, 1999), and qualitative reports that chronic pain patients treated with anterior cingulotomy continue to feel pain, but are less emotionally affected by it (Foltz and White, 1962, 1968).

More recently, rather than focusing on the brain activity related to the perception of pain, a number of functional neuroimaging studies have investigated the brain activity thought to be involved in mechanisms modulating pain, such as the top-down influence of nociceptive transmission at the level of the spinal cord (Bingel *et al.*, 2006; Tinnermann *et al.*, 2017), the changes in brain activity thought to be involved in central sensitization or placebo analgesia (Petrovic *et al.*, 2002; Wager *et al.*, 2004, 2007; Zambreanu *et al.*, 2005; Zubieta *et al.*, 2005; Lee *et al.*, 2008), the modulation of pain by cognitive control, emotions and attention (Brascher *et al.*, 2016). If successful, these approaches hold strong promise. Indeed, considering that pain-modulatory mechanisms might vary across individuals, being able to characterize their contribution at an individual level opens the prospect of being able stratify patients into functionally meaningful categories, to better understand the functional mechanisms contributing to the development and maintenance of chronic pain, and to potentially orient towards more personalized and effective treatment strategies taking into consideration individual ‘pain endophenotypes’ (Tracey, 2011). For example, functional neuroimaging could be used to assess the ability of an individual patient to engage descending inhibitory control mechanisms, or his susceptibility to sensitize when exposed to intense nociceptive stimulation. Such results would have immediate practical implications, as they might allow prediction of whether that patient will respond well to a specific treatment, or how likely it is that he will develop chronic pain after surgery. However, as recently emphasized by Denk *et al.* (2014), data currently supporting this view are still sparse and, most critically, do not allow one to infer causal relationships. Indeed, the use of proper controls is necessary to relate changes in brain activity to specific pain modulatory mechanisms. If brain structure A has a level of activity that relates positively to the amount of reduction in pain perception caused by a given treatment or experimental manipulation, this does not automatically mean that brain structure A is involved in generating the pain relief caused by the treatment. For example, if distraction from the painful stimulus is induced by performing a counting Stroop task (as in Bantick *et al.*, 2002), the increased neural activity observed in brain structure A during distraction could reflect brain processes engaged by the execution of the task, but independent of the processes responsible for the effect of the task on pain perception. Hence, observing that distraction from pain is associated with increased activity in brain structure A does not allow conclusion that this activity is ‘orchestrating the modulation of pain by attention’ (Bantick *et al.*, 2002). Finally, to be clinically useful, such neuroimaging measures must have predictive value at individual level. In this respect, the use of longitudinal designs is imperative to determine whether brain imaging can be useful to assess individual vulnerability to develop chronic pain. Although difficult to implement, a few studies have followed this line and generated encouraging results. For example, Baliki *et al.* (2012) recently showed that the state of cortico-striatal ‘reward-motivation circuits’ measured using functional MRI can predict the transition to chronic pain in patients with subacute low-back pain.

Along the same lines, a recent study from the same group compared the effects of placebo and duloxetine in patients with chronic pain, yielding striking results. Specific patterns of brain connectivity before receiving the treatment could predict which patients would be placebo responders, as well as the degree of analgesia that would be induced by both the placebo and the active agent (Tetreault *et al.*, 2016). Given their wide clinical implications in terms of patient stratification and drug development, these results demand replication. Still, they already hint towards the practical use of neuroimaging to predict response to treatment in chronic pain conditions.

In conclusion, it is important that further studies on brain biomarkers of pain and its modulation are conducted to test the biomarkers' generalizability, assess their performance at an individual level, and understand the reasons why they may correlate with pain or its modulation using longitudinal design studies and carefully designed control conditions. In any case, it is imperative to draw a clear line to distinguish between the clinical or experimental utility of a biomarker and its usefulness in achieving mechanistic insight. A biomarker can demonstrate good utility because it is able to identify clinically meaningful groups of patients with a high sensitivity and specificity (e.g. patients who will respond in a certain way to a specific treatment). However, this does not necessarily imply that the biomarker reflects directly the mechanisms that give rise to a given clinical pain condition, as the patterns of neural activation that allow discrimination between conditions might be entirely epiphenomenal.
